# Supplementary material for: First Report of Food Poisoning Due to Staphylococcal Enterotoxin Type B in Döner Kebab (Italy)
Source: Pathogens. 2023 Sep 6;12(9):1139. doi: 10.3390/pathogens12091139 (PMC10535471; doi:10.3390/pathogens12091139)
Supplement: Supplementary file 1 [file pathogens-12-01139-s001.zip › supplementary materials.pdf]

Table S1. Genome statistics calculated with Quast 5.0.2 [29]

Genome statistics	Food Handler 1_1	Food Handler 1_2	Food Handler 1_3	Food Handler 1_4	Food Handler 1_5	Food Handler 2_1	Food Handler 2_2	Food Handler 2_3	Food Handler 2_4	Food Handler 2_5	Kebab_1	Kebab_2	Kebab_3	Kebab_4	Kebab_5
**Genome fraction (%)**	94,534	94,309	94,527	94,579	94,484	94,438	94,283	94,477	94,335	94,454	94.39	94,492	94,315	94,346	94,349
**Total aligned length**	2,667,439	2,660,789	2,667,692	2,668,239	2,665,869	2,664,580	2,660,582	2,665,451	2,660,438	2,664,738	2,663,242	2,665,636	2,660,779	2,661,520	2,662,661
**# contigs (>= 1000 bp)**	23	18	20	18	20	24	23	20	20	23	19	20	19	20	18
**Largest contig**	868,268	1,195,986	1,196,036	1,195,853	1,195,853	868,084	851,962	1,195,985	868,258	1,139,865	1,304,178	997,159	868,154	1,195,985	1,196,036
**Total length**	2,770,767	2,764,463	2,770,715	2,772,167	2,769,460	2,768,260	2,763,300	2,769,826	2,764,805	2,768,492	2,767,099	2,769,691	2,764,139	2,764,883	2,765,714
**N50**	284,284	304,325	304,325	589,540	304,325	247,590	326,925	304,325	326,925	304,325	247,346	240,421	304,325	304,325	304,325
**GC (%)**	32.61	32.62	32.61	32.62	32.62	32.62	32.62	32.62	32.62	32.62	32.62	32.61	32.62	32.62	32.62

Table S2. Distance matrix obtained using SNP analysis with CSI Phylogeny 1.2 [12]

	Kebab 1	Kebab 2	Kebab 3	Kebab 4	Kebab 5	Food Handler 1/1	Food Handler 1/2	Food Handler 1/3	Food Handler 1/4	Food Handler 1/5	Food Handler 2/1	Food Handler 2/2	Food Handler 2/3	Food Handler 2/4	Food Handler 2/5	Outgroup
**Kebab 1**	0	0	5	4	6	6	3	21	3	19	9	4	4	22	20	2848
**Kebab 2**	0	0	5	4	6	6	3	21	3	19	9	4	4	22	20	2848
**Kebab 3**	5	5	0	5	5	5	2	20	2	18	8	3	3	21	19	2847
**Kebab 4**	4	4	5	0	6	6	3	21	3	19	9	4	4	22	20	2848
**Kebab 5**	6	6	5	6	0	6	3	21	3	19	9	4	4	22	20	2848
**Food Handler 1/1**	6	6	5	6	6	0	3	21	3	19	9	4	4	22	20	2848
**Food Handler 1/2**	3	3	2	3	3	3	0	18	0	16	6	1	1	19	17	2845
**Food Handler 1/3**	21	21	20	21	21	21	18	0	18	8	24	19	19	11	9	2851
**Food Handler 1/4**	3	3	2	3	3	3	0	18	0	16	6	1	1	19	17	2845
**Food Handler 1/5**	19	19	18	19	19	19	16	8	16	0	22	17	17	7	5	2849
**Food Handler 2/1**	9	9	8	9	9	9	6	24	6	22	0	7	7	25	23	2851
**Food Handler 2/2**	4	4	3	4	4	4	1	19	1	17	7	0	2	20	18	2846
**Food Handler 2/3**	4	4	3	4	4	4	1	19	1	17	7	2	0	20	18	2846
**Food Handler 2/4**	22	22	21	22	22	22	19	11	19	7	25	20	20	0	8	2852
**Food Handler 2/5**	20	20	19	20	20	20	17	9	17	5	23	18	18	8	0	2850
**Outgroup**	2848	2848	2847	2848	2848	2848	2845	2851	2845	2849	2851	2846	2846	2852	2850	0

min: 0 max: 2852
